# Efficacy of repeated peripheral magnetic stimulation on upper limb motor function after stroke: a systematic review and meta-analysis of randomized controlled trials

**DOI:** 10.3389/fneur.2025.1513826

**Published:** 2025-04-03

**Authors:** Defu Liao, Ziyan He, Shichang Yan, Qipei Ji, Yuanlin Li, Yuyuan Tu, Zihao Zhou, Shuangchun Ai

**Affiliations:** ^1^School of Health and Rehabilitation, Chengdu University of Traditional Chinese Medicine, Chengdu, China; ^2^Department of Rehabilitation, Mianyang Hospital of Traditional Chinese Medicine, Mianyang, China

**Keywords:** repetitive peripheral magnetic stimulation, motor function, stroke, upper limb, meta-analysis

## Abstract

**Background:**

Post-stroke patients with upper motor neuron lesions have limited motor function in the upper limbs, and spasticity occurs in the limbs, thus affecting functional recovery and activities of daily living. Repetitive peripheral magnetic stimulation (rPMS) is a non-invasive treatment often used in clinical rehabilitation. Recent studies have shown that it can reduce spasticity and improve motor function in patients.

**Objective:**

This study aimed to evaluate the effectiveness of rPMS on upper limb motor function and spasticity in stroke patients by meta-analysis.

**Materials and methods:**

Randomized controlled trials (RCTs) of rPMS in post-stroke patients were searched in PubMed, Embase, Cochrane Library, Web of Science, and Clinical Trials. Databases from the date of creation to 25 August 2024 were evaluated using the Cochrane Collaboration tool. Methodological quality was assessed using the Cochrane Collaboration tools, and meta-analyses were performed using RevMan (version 5.4) and Stata (version 14.0).

**Results:**

A total of 8 studies were included. RPMS improved patients’ FMA-UE scores compared with controls (MD = 3.34, 95% CI = [0.53, 6.15], *p* = 0.02 < 0.05). RPMS also reduced spasticity (MD = −0.66, 95% CI = [−1.16, −0.15], *p* = 0.01 < 0.05) and increased patients’ ability to live independently (MD = 0.85, 95% CI = [0.19, 1.51], *p* = 0.01 < 0.05). Subgroup analyses showed that the efficacy of treatment frequency ≤ 20 Hz was better than that of frequency > 20 Hz; the treatment time using 15–20 min was more effective than using 30 min; and the application of round coil treatment was more effective than other types of coils.

**Conclusion:**

The results suggest that if rPMS is used in post-stroke patients, their upper limb motor function and spasticity may improve. However, the number of studies is small, and further research is needed to extend the current analysis results.

**Systematic review registration:**

https://www.crd.york.ac.uk/prospero/, CRD42024584040.

## 1 Introduction

Stroke, being one of the more prevalent diseases globally, causes severe distress to patients and their families in terms of quality of life, finances, and man-hours. Globally, stroke is the second leading cause of death, accounting for 11.6% of all deaths (1). At the same time, the incidence of stroke is getting younger, which may be related to modern advanced neuroimaging or the dietary and work habits of young people (2). A range of complications can exist after stroke, including dysphagia, impaired consciousness, upper limb motor dysfunction, and cognitive dysfunction (3). If not treated effectively, upper limb motor dysfunction will seriously affect the patient’s daily life activities and cause inconvenience.

The treatment of upper limb motor dysfunction after stroke is based on adaptation or plasticity of the brain after the injury through the practice of specific tasks, medications, robotic trainers, and other methods of enhancing motor learning (4). Improvements in motor function can be achieved using Constraint Induced Movement Therapy (CIMT), which is an operant approach to progressively shape functionally more useful movements using a set of standardized tasks for reaching, grasping, and pinching (5) or by injecting Botulinum Toxin Type A (BoNT-A) to reduce spasticity in the patient’s limbs (6). In recent years, non-invasive stimulation (neuromuscular electrical stimulation, transcranial direct current stimulation, repetitive transcranial magnetic stimulation, and transcutaneous electrical nerve stimulation) has been applied to improve motor function after stroke (7, 8). However, we found that the use of repeated peripheral magnetic stimulation (rPMS) was rare due to unknown parameters and uncertainty about the stimulation site (9).

RPMS is the use of time-varying pulsed magnetic fields of a certain intensity to stimulate excitable tissues, thereby generating induced currents within the tissues, which pass through the nerve cell membranes and enter the axons, resulting in a change in cell membrane potential (10). When the intensity of the stimulus exceeds the cellular threshold, it causes the cell to depolarise to generate an action potential, which in turn causes the muscle to contract (11). Different parameters are applied to reduce pain or promote sensorimotor recovery (12, 13). Impairment of proprioceptive inputs may lead to slower recovery of motor function after stroke (14), one way to restore motor function in patients seems to be to enhance their proprioceptive stimulation. RPMS activates the remodeling of neural tissue in the brain by stimulating proprioceptive inputs, which in turn improves motor function (15). RPMS provides proprioceptive input to the CNS (central nervous system) in two different ways (16), one is direct activation: direct activation of sensorimotor nerve fibers through cis and transduction. The other is indirect activation: indirect activation through mechanoreceptors (class Ia, Ib, and II muscle fibers) during muscle contraction and relaxation. However, the preferential recruitment of cutaneous and proprioceptive afferents over nerves and muscles by rPMS remains controversial (9). There is evidence that the use of rPMS reduces spasticity on the affected side and increases sensory function on the hemiplegic side of the patient (17, 18). RPMS is a painless, non-invasive treatment that has negligible side effects. Suzuki et al. (19) applied rPMS to a male Wistar rat animal model and found that the use of rPMS may not produce damage to the muscles at the application site. Meanwhile, compared with conventional electrical stimulation, rPMS has the advantages of deeper depth and stronger stimulation force.

Although a meta-analysis by Momosaki et al. (20) showed improvement in upper limb spasticity in patients treated with rPMS, there was no statistically significant improvement in upper limb motor function in patients. This meta-analysis aimed to derive the feasibility of rPMS to improve upper limb motor function by analyzing the improvement of upper limb motor function in patients treated with rPMS as well as subgroup analyses at different frequencies, with different coil models, time of stimulation use, and length of post-stroke disease cycle, and to conclude on potentially appropriate therapeutic parameters.

## 2 Methods

This study was registered with PROSPERO, registration number CRD42024584040. It was conducted according to the Preferred Reporting Items for Systematic Evaluation and Meta-Analyses (PRISMA) (21).

### 2.1 Search strategy

From the time of library construction to 25 August 2024, two review authors independently searched PubMed, Embase, Cochrane Library, Web of Science, and Clinical Trials. The search terms included repetitive peripheral magnetic stimulation, magnetic stimulation, stroke, acute ischemic stroke, upper limb function, motor function, and randomized controlled trials. The search terms are documented in detail in the Supplementary Appendix Table 4. In addition, we manually checked all reference lists of the retrieved papers and asked experts for any potentially relevant studies.

### 2.2 Inclusion and exclusion criteria

Studies were selected if they met the following inclusion criteria: ① the subjects were post-stroke upper limb motor dysfunction; ② the experimental group was treated with rPMS on top of the control group; ③ these studies used widely recognized scales such as the upper-extremity motor section of the Fugl-Meyer Motor Assessment Scale (FMA-UE), Modified Ashworth scale (MAS), and so on; ④ the study design was a randomized controlled study.

Studies that met the following criteria were excluded: ① conference reports, abstracts, animal tests, and replicated studies; ② objective data were missing or could not be extracted, and the full text could not be obtained by contacting the corresponding authors; ③ other studies in which non-motor function endpoints interfered with the observation of efficacy; and ④ non-English literature.

### 2.3 Study selection

Endnote X9 was used to manage the search records. Two evaluators independently screened titles and abstracts of potentially eligible studies against the inclusion criteria after removing duplicate results. They then read the full text of potentially eligible studies to determine the final literature for inclusion. If there was disagreement, a third reviewer was invited to discuss and make a decision.

### 2.4 Data extraction

Two evaluators used the data extraction form to extract the required data from the included studies. In case of disagreement, a third researcher resolved the dispute. The main elements of data extraction were as follows: ① First author of the literature, time of publication, frequency of device application, treatment site, intensity of treatment, duration of treatment, and type of coil ② Treatment methods used in the control group ③ Indicators and data related to the outcome. ④ For literature that reported data only in the form of images, we used GetData Graph Digitizer software to extract data from the images.

### 2.5 Risk of bias assessment

Using the Cochrane Risk of Bias Assessment (22), two evaluators independently assessed the risk of bias. The tool categorizes studies into three categories based on their risk of bias: low, high, or unclear. These categories were selection bias (random sequence generation and allocation concealment), implementation bias (blinding of subjects and staff), detection bias (blinding of outcome assessment), attrition bias (incomplete outcome data), reporting bias (selective reporting bias), and other biases. In the event of a dispute, a third reviewer was introduced.

### 2.6 Certainty of the evidence

The rating of recommendations is grounded in the GRADE (Grading of Recommendation Assessment, Development, and Evaluation) methodology, in which the certainty of the evidence is categorized as “very low,” “low,” “medium,” or “high” (23). The quality of randomized controlled trials is high, while the quality of observational studies is low. Research quality can be lowered by five factors: limitations, inconsistency, indirectness, imprecision, and publication bias (24).

### 2.7 Data analysis

The statistical significance threshold was set at *p* < 0.05, and the data were integrated with RevMan 5.4 and Stata 14.0. The outcomes of this study were continuous variables, and for each effect size, the researchers calculated 95% confidence intervals (CIs). For outcomes assessed using the same scale, data were combined using mean difference (MD), and descriptive analyses were used for effect sizes that could not be combined. Depending on the test of heterogeneity, the treatment was treated using either a fixed-effects model or a random-effects model. A fixed-effects model was used when the test of heterogeneity for the included studies was *p* > 0.05 and *I*^2^ < 50%; a random-effects model was used when *p* < 0.05 and *I*^2^ ≥ 50%. Subgroup analyses were performed according to different frequencies, different models of coils, different sites of stimulation, and length of disease cycle. Forest plots were used to display the combined estimates according to forest plots. Sensitivity analyses were used to explore the causes of heterogeneity. Funnel plots were used to assess potential publication bias (25, 26).

## 3 Results

### 3.1 Selection and inclusion of studies

**Figure 1:**
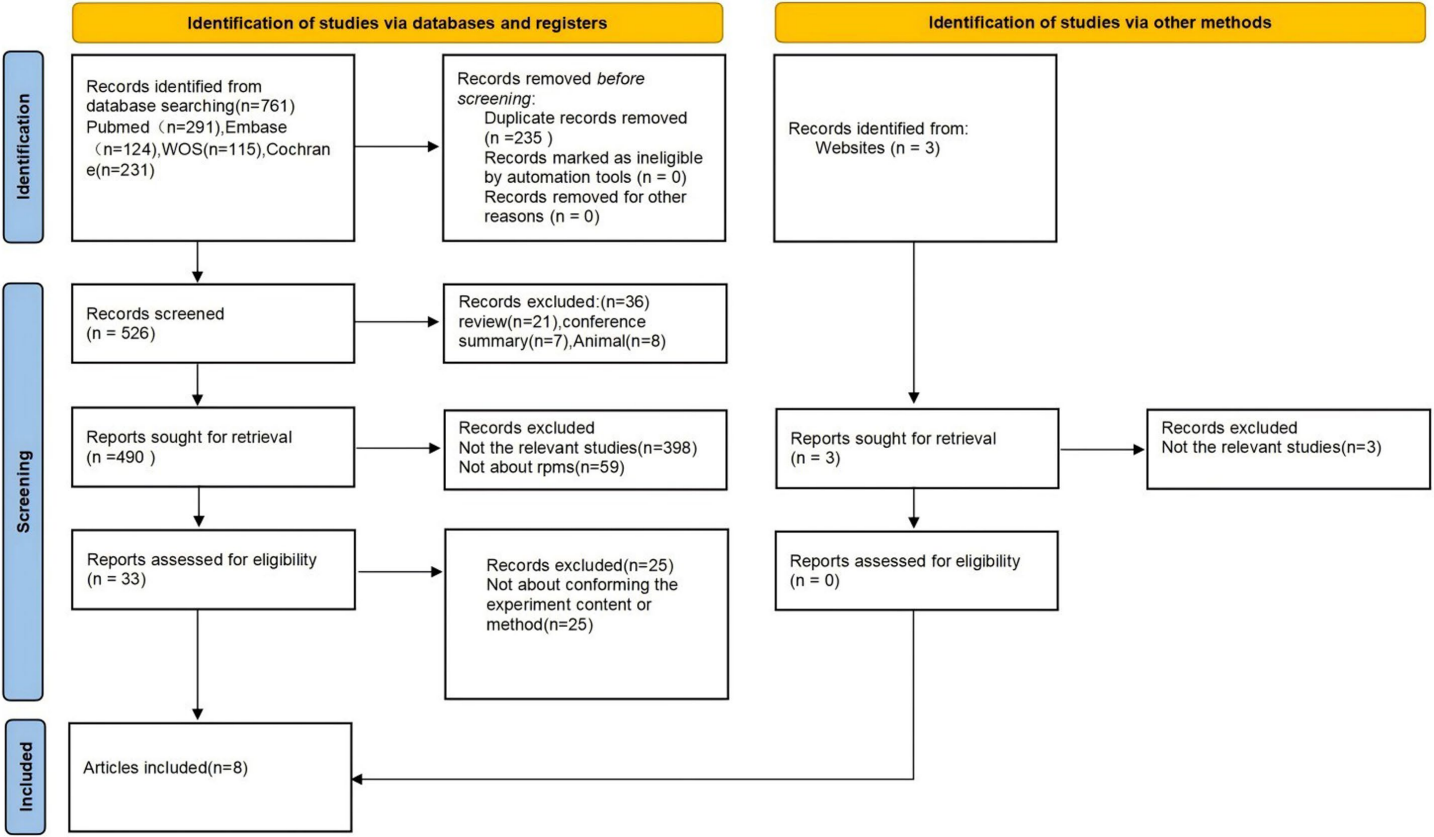
Project for Reporting of Systematic Evaluations and Meta-Analyses (PRISMA) flowchart.

An initial search of 764 studies was conducted through the above databases. Eight studies were identified through multiple screening steps (17, 18, 27–32) for meta-analysis. Examples include exclusion of duplicates, selection of literature types, systematic review of title and abstract competitions, and review of full text (Figure 1).

### 3.2 Characteristics of the included studies

A total of eight studies were included in this study. RPMS differed in terms of the application site, treatment frequency, treatment intensity, number of pulses, on–off ratio, treatment duration, and coil type. Five studies (17, 18, 27, 30, 32) stimulated more than two groups of upper limb muscles, one study (28) stimulated the triceps brachii muscle only, and the remaining two studies (29, 31) illustrated that the stimulation site was the axilla with the affected arm. According to the treatment frequency of the repetitive peripheral magnetic stimulation application, four studies (17, 28, 29, 31) had a treatment frequency of less than or equal to 20 Hz, and four studies (18, 27, 30, 31) had a treatment frequency of more than 20 Hz. Concerning the intensity of the treatment, three studies (28–30) used it by the percentage of the maximum output value of the treatment apparatus, and three studies (18, 27, 32) performed the treatment according to the intensity at which movement could occur in the wrist at rest, one study (31) performed the treatment according to the individualization of the patient, and one study (17) did not mention the intensity of the treatment. Regarding the on–off ratio, two studies (31, 32) had a treatment time of 2 s and a rest of 8 s, while the remaining six studies (17, 18, 27–30) had different on–off ratios. Regarding the duration of each application of repetitive peripheral magnetic stimulation treatment, four studies (17, 27, 29, 32) had treatment durations greater than 20 min, and three studies (18, 28, 30) had treatment durations less than or equal to 20 min, whereas one study (31) did not mention it. Regarding treatment duration, six studies (17, 18, 28, 29, 31, 32) had a treatment duration of less than or equal to 2 weeks, one study (27) had a treatment duration of more than 2 weeks, whereas one study (30) determined a treatment duration based on the time of the patient’s transfer to the hospital. Regarding the type of coils used in the treatment devices, two studies (18, 27) used butterfly coils, three studies (27, 28, 30) used circular coils, three studies (29, 31, 32) used figure-of-eight toroidal coils, whereas one study (17) used parabolic coils. The specific basic characteristics of the included studies are shown in Supplementary Appendix Table 1.

### 3.3 Analysis of quality evaluation methods

**Figure 2:**
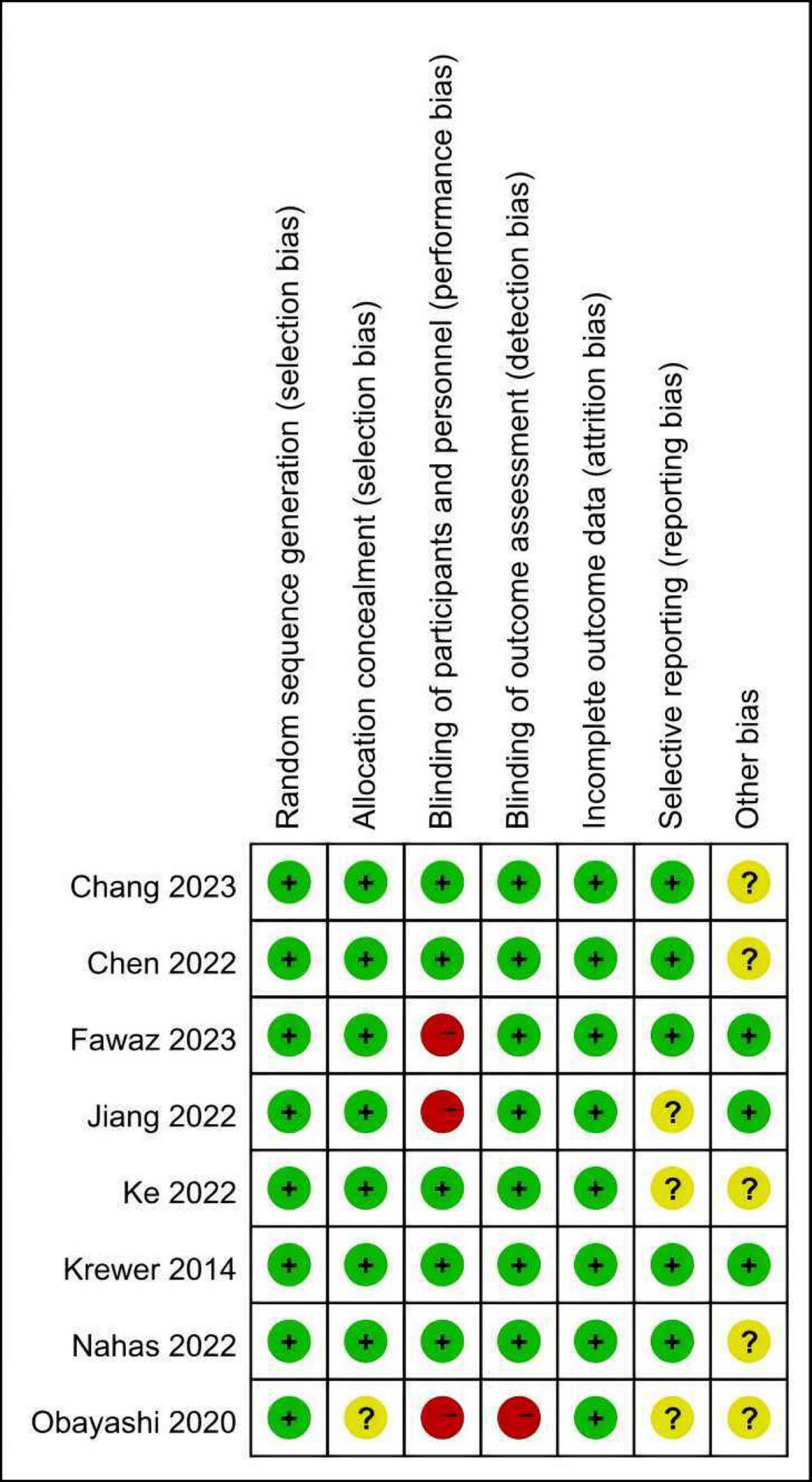
Risk of bias summary: review authors’ judgment of each risk of bias item for each included study.

**Figure 3:**
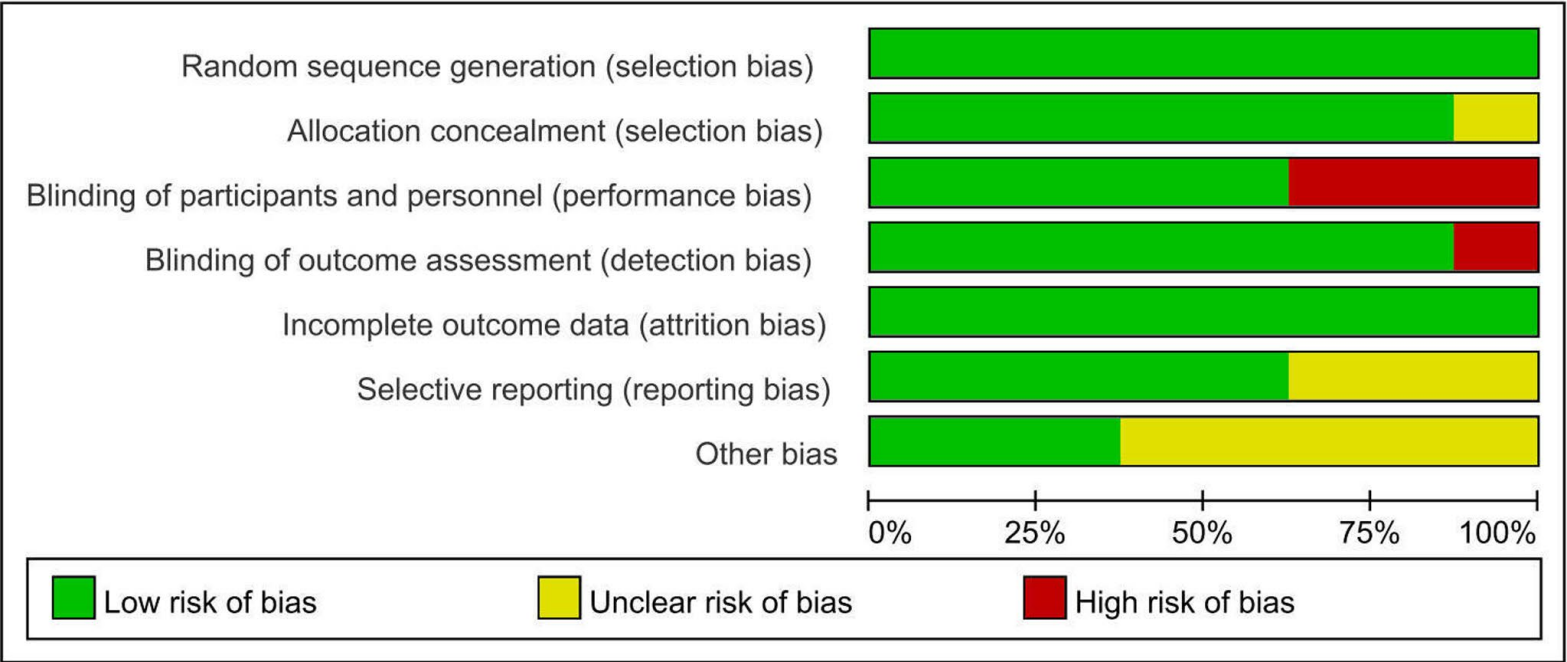
Risk of bias plot: Review authors’ judgment for each risk of bias item, expressed as a percentage across all included studies.

The risk of bias graph for each included study is shown in Figure 2, and the percentages for each study are shown in Figure 3. All of the studies mentioned random allocation sequences and allocation concealment; one study was assessed as having some problems because details of allocation concealment were not mentioned, and seven studies reported details of allocation concealment. Five studies mentioned blinding, and although these studies used different types of interventions, subjects, and staff were not informed of whether they were in the experimental or the control group. One study did not mention blinding the blinding of subjects and staff. In addition, three studies may have had incomplete outcome data and selective reporting. In one study, some data were extracted from images using GetData Graph Digitizer software, which may have been biased.

### 3.4 Results of the meta-analysis

#### 3.4.1 FMA-UE analysis

**Figure 4:**
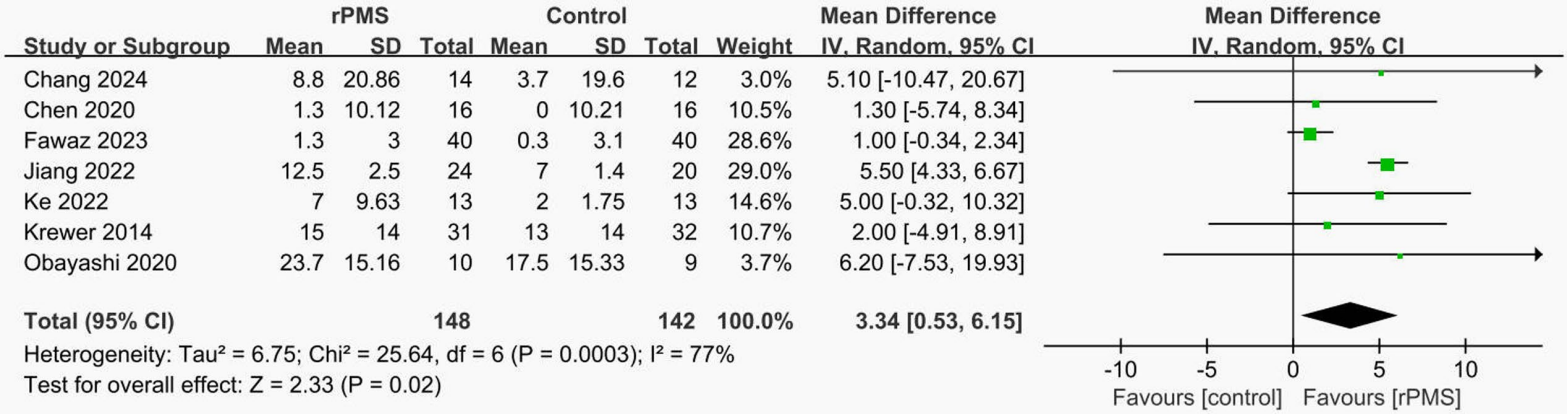
Forest plot of FMA-UE outcome.

**Figure 5:**
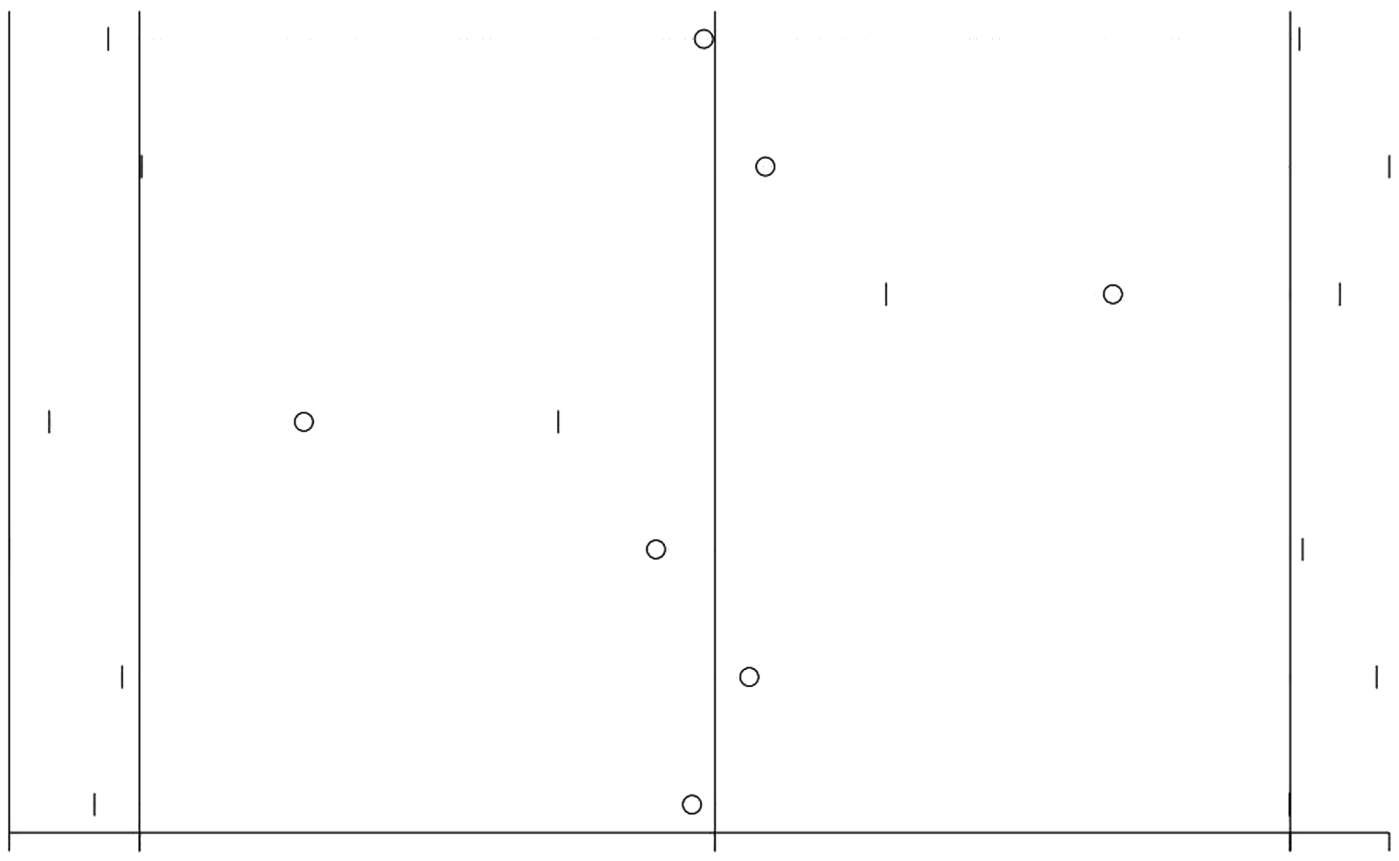
Sensitivity analysis of FMA-UE.

In total, seven studies (17, 18, 27–32) reported on FMA-UE, involving 290 patients. The results showed that rPMS improved patients’ FMA-UE scores (MD = 3.34, 95%CI = [0.53, 6.15], *p* = 0.02 < 0.05) (Figure 4). Sensitivity analyses showed that excluding each study did not affect the stability of our trial (Figure 5).

##### 3.4.1.1 Subgroup analyses

Subgroup analyses of rPMS for improving patients’ FMA-UE scores and improving upper limb motor ability showed (as shown in Supplementary Appendix Table 2) that adults under 55 years of age showed better improvement in motor function than adults over 55 years of age; treatment frequency ≤ 20 Hz was more efficacious than frequency > 20 Hz; treatment duration using 15–20 min was more effective than using 30 min; treatment of stroke onset >40 days was slightly more effective; treatment duration 2 weeks showed the best results; and application of round coil treatment was more effective than other types of coils was more effective.

##### 3.4.1.2 Publication bias

**Figure 6:**
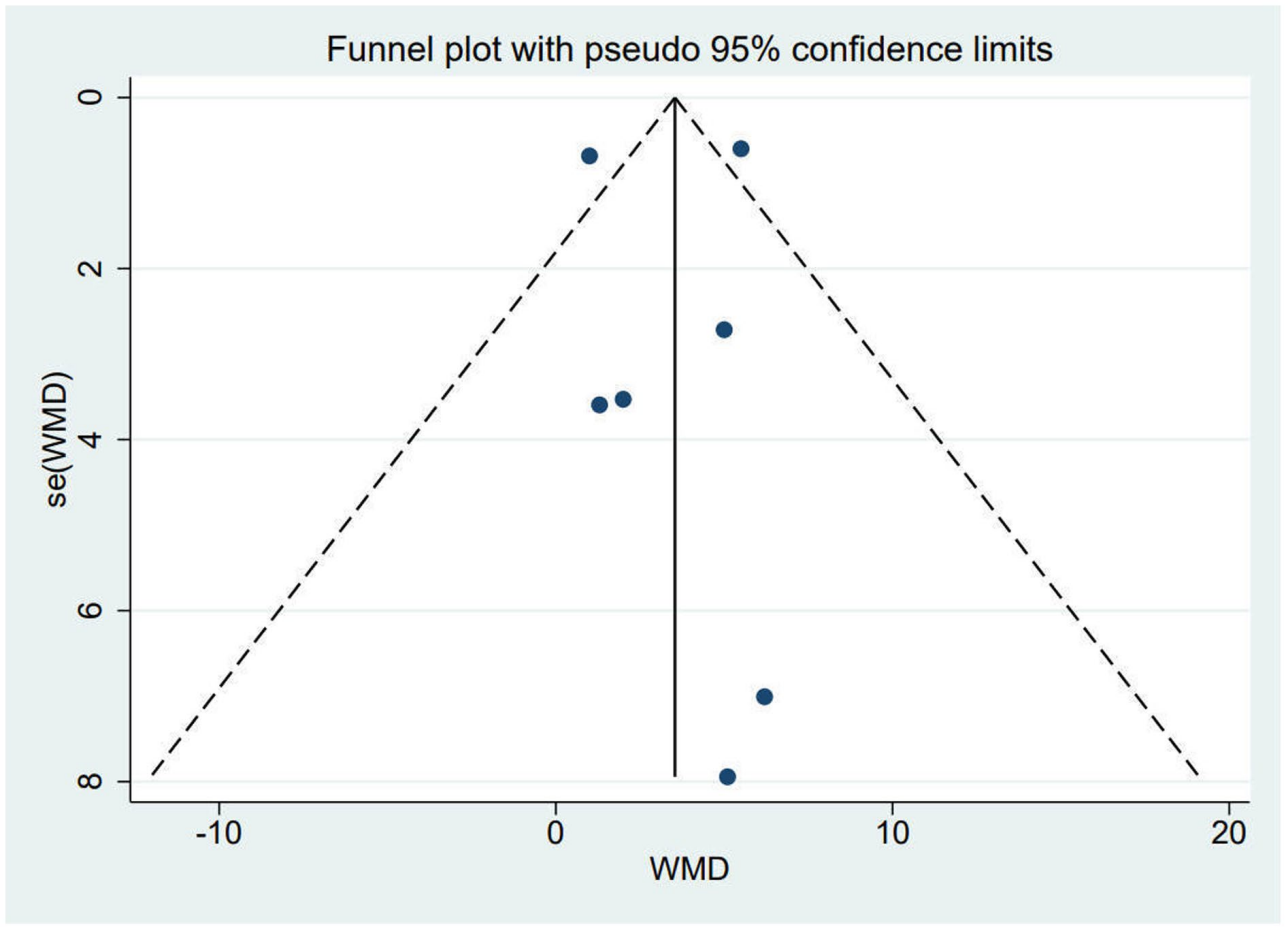
Funnel plot of FMA-UE.

As shown, we checked for publication bias by performing funnel plots as well as Egger’s test on the FMA-UE scores of the seven study groups. The funnel plot showed basic symmetry and *p* = 0.93 in the Egger test, indicating no publication bias (*p* > 0.05) (Figure 6).

#### 3.4.2 Analysis of FIM

**Figure 7:**
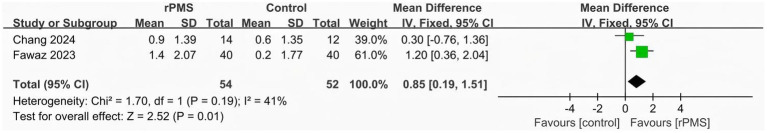
Forest plot of FIM outcome.

A total of two studies (27, 31) discussed FIM (functional independence measure), involving 106 patients. A fixed-effects meta-analysis showed that FIM scores were improved in the rPMS group compared to the control group. (MD = 0.85, 95% CI = [0.19, 1.51], *p* = 0.01 < 0.05) (Figure 7).

#### 3.4.3 Analysis of MAS

**Figure 8:**
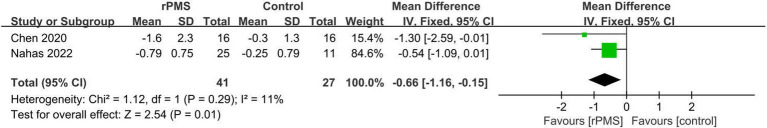
Forest plot of MAS outcome.

A total of two studies (17, 32) discussed MAS, involving 68 patients. Fixed-effects meta-analyses showed a significant reduction in MAS scores in the rPMS group compared to the control group (MD = −0.66, 95% CI = [−1.16, −0.15], *p* = 0.01 < 0.05) (Figure 8).

### 3.5 Certainty of the evidence

The GRADE results are presented in the Supplementary Appendix Table 3. The level of evidence certainty was rated as “moderate” for FMA-UE. The level of evidence certainty was rated as “low” for MAS and FIM. The leading causes of the sample size of the included study were too small, and the confidence interval was too broad.

## 4 Discussion

### 4.1 Effects of rPMS

A total of eight randomized controlled trials were included in this study to systematically evaluate the use of rPMS in patients with upper limb dysfunction after stroke. Compared with sham stimulation or no treatment, rPMS may be a treatment that can improve upper limb motor function, improve spasticity, and lead to a higher level of independent living in post-stroke patients.

Two studies (17, 18) in this review mentioned the Modified Tardieu Scale (MTS) to measure spasticity in patients (33), but a meta-analysis of MTS could not be performed because the two studies used different MTS protocols. However, one of the studies (17) reported a reduction in motor spasticity after treatment with rPMS, whilst one study (18) also reported a long-term effect on reducing spasticity in elbow extensors after 2 weeks of treatment but a limited effect on overall spasticity in the upper limb. One meta-analysis (34) showed that there was insufficient evidence to suggest that rPMS could change patients’ motor function. However, in this paper, seven studies (17, 18, 27–31) used the FMA-UE to evaluate upper limb motor function, and the overall meta-analysis showed that rPMS was beneficial in improving patients’ upper limb motor function. A study (18) showed that there was no significant improvement in motor function after rPMS intervention on the extensor and flexor muscles of the upper arm. This may be because rPMS was applied to different areas or at different frequencies.

Two studies (27, 31) used the FIM scale to measure upper limb self-care in patients after treatment. Fawaz et al. (27) demonstrated that rPMS combined with occupational therapy for 3 weeks resulted in significant improvement in FIM scores. Another study (31) showed that after 2 weeks of the combination of rPMS with occupational and physiotherapy, there was a significant improvement in the experimental and control groups, but there was no difference in change scores between the two groups. There were two studies (17, 32) on the use of MAS to measure spasticity in patients before and after treatment, in which the application of sham stimulation improved spasticity in the upper limbs of patients with a significant reduction in MAS in the rPMS group compared to the rPMS group, which is consistent with the results of a meta-analysis (35).

### 4.2 Possible mechanisms of improved motor function and spasticity with rPMS

RPMS enhances proprioceptive inputs by generating deep muscle stimulation, stimulating the brain with deep muscle contractions to activate and induce brain plasticity (36), which is the basis of human motor learning (37) and enables the integration of proprioception into somatic movements to co-ordinate motor functions. RPMS induces activation of the frontoparietal sensory-motor cortex and changes in intracortical and corticospinal motor excitability. Gallasch et al. (38) found that rPMS intervention promoted intracortical facilitation and increased the recruitment profile of motor-evoked potentials in the radial carpal flexors and enhanced activation of sensorimotor areas in the contralateral brain. This is due to the two pathways of rPMS that induce proprioceptive inputs to the brain, leading to neuromodulation of the brain. Some studies (39) also found that this proprioceptive input traveled along the upward sensory pathway to the primary sensory cortex (S1) in the parietal region, which then facilitated the reorganization of the primary motor cortex (M1) area through the functional and structural connections between the S1 area and M1 area, thus improving the patient’s motor function.

In the weeks following a stroke, patients experience an increase in muscle tone due to damage to the pyramidal tract and, in severe cases, spasticity. Generally, increased muscle tone is accompanied by impaired proprioceptive input (40). RPMS was found by Krause et al. (41) to be a potential therapeutic option for spasticity, and it also promotes modulation and plasticity processes in the CNS. RPMS promotes proprioceptive inputs that lead to plasticity in the M1 area and improved sensorimotor function (42), and the principle of improving spasticity may be similar to that of improving motor function. RPMS promotes the reorganization of M1 areas, which in turn improves motor function. RPMS can also improve spasticity by inducing a certain degree of muscle morphology and increasing muscle blood flow, which can result in a certain softening of muscle-stimulated tissues (43, 44).

### 4.3 Effect of rPMS dose and coil type

The use of rPMS involves the selection of different parameters as well as coils. Suitable parameters are a guarantee of safety during treatment, and different coils connected to the magnetic stimulation will also have different effects. For example, figure-of-eight coils are suitable for precise localization of pain triggers (9), trigger points (45), or motor points (46) in a given area, whereas round coils can stimulate deeper and larger areas of painful muscles and are used in nerve roots (47) or the back (48). In our subgroup analysis, the therapeutic effect was better with the use of the round coil, which was able to effectively act on deep muscle groups and promote proprioceptive inputs to achieve therapeutic effects (13).

There is no definitive, sufficient evidence for stimulation frequency to suggest that low frequencies are inhibitory and high frequencies are excitatory. In a study by Struppler (36) and others, a more lasting improvement in spasticity and motor control was found with 20 Hz rPMS, which is consistent with the ideas presented in this paper. Regarding the null ratio, some studies used continuous stimulation, and others used intermittent stimulation, and all the studies included in this paper used an ON/OFF (intermittent stimulation) protocol, but their specifics were different. In a review (9), it was mentioned that the mean ratio of on–off ratios in studies where rPMS was applied to sensorimotor injuries was 3.9 ± 2.2%. However, there is no reason to support the difference between the studies. The longer duration of OFF in the intermittent protocol in this paper may be related to coil overheating. Continuous stimulation fatigues the muscles, whereas intermittent stimulation physiologically contracts/relaxes the muscles and generates a large flow of proprioception into the sensorimotor network via the upward pathway, thus affecting neuroplasticity mechanisms (49, 50). There are generally two choices of treatment intensity: one is the threshold of muscle contraction, and the other is the maximum output of the therapeutic apparatus. RPMS the choice of treatment intensity depends on the depth of the target structure and the afferent nerves recruited. Therefore, different choices are used to affect sensorimotor deficits. In this study, shorter (15–20 min) versus longer (30 min) duration was used for subgroup analysis, showing that shorter duration achieved better therapeutic results and can improve spasticity and cortical motor control, a point of view that is in line with previous studies (51). However, in clinical treatment, the duration of the stimulation sequence, the interval between sequences, and the frequency of stimulation need to be considered in light of the patient’s functional condition, the therapeutic purpose, and the patient’s tolerance level.

### 4.4 Limitations of this study

The limitations of this systematic evaluation are as follows: ① The two secondary endpoints of the meta-analysis were based on small sample sizes, and it is unlikely that sufficient effects were obtained to confirm the effect sizes of the corresponding endpoints (52). ② Blinding was not mentioned in one study, and in one study, the data were extracted from images, which may have led to bias. ③ The site, frequency, duration, and on–off ratio of rPMS treatment were different in the included studies, leading to significant heterogeneity among the studies.

## 5 Conclusion

The results suggest that if rPMS is used in post-stroke patients, their upper limb motor function spasticity may improve. Although it was not possible to obtain specific parameters, we can consider using rPMS with 20 HZ and a circular coil type for 15–20 min. The intensity of the treatment, as well as the on/off ratio, needs to be considered according to the patient’s tolerance level and the specifications of the treatment apparatus. There are no articles that give a suitable range for rPMS, and further studies are needed to extend the current analysis to include more patients and incorporate standard outcome measures to explore the optimal protocol for rPMS.

## Data Availability

The original contributions presented in the study are included in the article/Supplementary material, further inquiries can be directed to the corresponding author.
