# Supplementary material for: Efficacy of repeated peripheral magnetic stimulation on upper limb motor function after stroke: a systematic review and meta-analysis of randomized controlled trials
Source: Front Neurol. 2025 Apr 3;16:1513826. doi: 10.3389/fneur.2025.1513826 (PMC12003123; doi:10.3389/fneur.2025.1513826)
Supplement: Supplementary file 1 [file Table_1.DOCX]

| **Included**  **study** | **Participants**  **（n）** | **Sex**  **(male/female)** | **Mean Age**  **（Mean±SD/**  **Median[IQR]）** | **Time post-stroke（Mean±SD/**  **Median[IQR]）** | **targeted points** | **Frequency**  **Intensity**  **No of pulses** | **On/**  **Off**  **(s)** | **Time /**  **Treatment duration** | **Coil**  **type** | **CG**  **Intervention** | **Additional**  **Intervention** | **Outcomes**  **Not mentioned** |
| --- | --- | --- | --- | --- | --- | --- | --- | --- | --- | --- | --- | --- |
| **Chen 2020** | **n=32** | **23/9** | **EG: 49.0±18.2**  **CG: 45.6±8.3** | **EG:37.4±42.0 month**  **CG:22.8±26.7 month** | **from shoulder adductors to extensors**  **from elbow flexors to extensors**  **from wrist flexors to extensors** | **5Hz/20HZ**  **Not mentioned**  **750/5100** | **3/1**  **1.5/1** | **30min**  **One time** | **parabola coil** | **Sham** | **——** | **FMA-UE**  **MAS**  **MTS** |
| **Fawaz 2023** | **n=80** | **56/24** | **mean age**  **57.33±10.67** | **from 6**  **to 60 weeks** | **Shoulder abductors**  **Elbow extensors**  **Wrist extensors**  **Supinator muscle** | **30HZ**  **Above 10%MCT**  **4500** | **5/1** | **30min**  **3 weeks** | **round coil**  **butterfly coil** | **Sham** | **Occupational**  **therapy** | **FMA-UE**  **FIM** |
| **Jiang 2022** | **n=44** | **27/17** | **EG:54.6±10.89**  **CG:56.09±10.89** | **EG:13.81±2.51 weeks**  **CG:14.45±3.33 weeks** | **triceps brachii** | **20HZ**  **15%-30%MSO**  **2400** | **0.5/2** | **20min**  **2weeks** | **round coil** | **untreated** | **physical therapy** | **FMA-UE** |
| **Ke 2020** | **n=26** | **14/12** | **EG:58(46.5-63.0)**  **CG:56(46.5-61.5)** | **EG:17(8-42.5) days**  **CG:16(4.5-22) days** | **the axilla** | **20HZ**  **40-60%MSO**  **1800** | **1/19** | **30 min**  **10 days** | **figure-of-eight coil** | **Sham** | **Conventional treatments** | **FMA-UE**  **MRC** |
| **Krewer 2014** | **n=63** | **25/38** | **EG:55±13**  **CG:54±13** | **EG:26±71 weeks**  **CG:37±82 weeks** | **Extensors and flexors**  **of the upper arm** | **25HZ**  **Above 10%MCT**  **5000** | **1/2** | **20min,2times**  **/day**  **2 weeks** | **butterfly coil** | **Sham** | **occupational therapy** | **FMA-UE**  **MTS**  **BI** |
| **Obayashi 2020** | **n=19** | **13/6** | **EG: 64.3±13.1**  **CG: 72.3±10.7** | **EG:9.2±4.4 days**  **CG:5.8±2.2 days** | **extensor digitorum communis**  **extensor carpi radialis**  **flexor digitorum superficialis**  **triceps brachii**  **biceps brachii**  **anterior or middle head of deltoid** | **30HZ**  **70%MSO**  **Not mentioned** | **2/2** | **15–20 min**  **until**  **transfer** | **round** | **SC** | **SC** | **FMA-UE**  **WMFT-FAS**  **BBT** |
| **Chang 2024** | **n=28** | **15/13** | **EG: 51.4±12.1**  **CG: 55.6±10.3** | **Not mentioned** | **patient’s arm** | **5HZ**  **individually adjusted**  **Not mentioned** | **2/8** | **Not mentioned**  **2 weeks** | **figure-of-eight** | **Sham** | **physical**  **therapy**  **occupational therapy**  **iTBS** | **FMA-UE**  **ARAT**  **FIM** |
| **Nahas 2022** | **n=36** | **27/9** | **EG: 47.88±14.8**  **CG:41.60±14.9** | **Not mentioned** | **biceps brachii and wrist/**  **finger flexor group** | **50HZ**  **Above MCT**  **600** | **2/8** | **1600 s;**  **Daily, 8 days** | **figure-of-eight** | **Sham** | **——** | **MAS** |

**EG:experimental group; CG:control group; MCT:muscle contraction threshold; MCO:maximal stimulator output SC:standard care; FMA-UE:the upper-extremity motor section of the Fugl-Meyer Motor Assessment Scale;BI:Barthel Index**

**MAS:Modified Ashworth scale MTS:Modified Tardieu Scale; MRC:Medical Research Council scale；BBT:box and block test；ARAT:Action ResearchArm Test；FIM:Functional Indenpendence Measure; iTBS:intermittent Theta Burst Stimulation**

**Characteristics of the included literature**
