# Supplementary material for: Efficacy of repeated peripheral magnetic stimulation on upper limb motor function after stroke: a systematic review and meta-analysis of randomized controlled trials
Source: Front Neurol. 2025 Apr 3;16:1513826. doi: 10.3389/fneur.2025.1513826 (PMC12003123; doi:10.3389/fneur.2025.1513826)
Supplement: Supplementary file 2 [file Table_2.DOCX]

| **Subgroup** | **Number of**  **studies** | **patients**  **（EG/CG）** | **Overall effect size (95% Cl)p** | | **Heterogeneity /p** | |
| --- | --- | --- | --- | --- | --- | --- |
| Age | | | | | | |
| ≤55 | 3 | 61/60 | 1.97 [-2.73,6.67] | 0.41 | 0% | 0.91 |
| >55 | 3 | 63/62 | 1.95[-0.01,2.57] | 0.05 | 21% | 0.28 |
| Frequency | | | | | | |
| ≤20HZ | 3 | 51/45 | 5.47 [4.33,6.62] | <0.00001 | 0% | 0.98 |
| >20HZ | 3 | 81/81 | 1.08 [-0.22,2.39] | 0.1 | 0% | 0.74 |
| Treatment time | | | | | | |
| 15-20min | 3 | 65/61 | 5.41 [4.25,6.56] | <0.00001 | 0% | 0.62 |
| =30min | 3 | 134/130 | 1.29 [-0.12,2.69] | 0.07 | 2% | 0.36 |
| Time post-stroke | | | | | | |
| ≤40days | 2 | 23/22 | 5.16 [0.20,10.12] | 0.04 | 0% | 0.87 |
| >40days | 3 | 71/68 | 5.29 [4.15,6.44] | <0.00001 | 10% | 0.03 |
| Treatment duration | | | | | | |
| 2weeks | 3 | 69/64 | 5.40 [4.25,6.55] | <0.00001 | 0% | 0.62 |
| 3weeks | 1 | 40/40 | 1.00 [-0.34,2.34] | 0.14 | - | - |
| 10days | 1 | 13/13 | 5.00 [-0.32,10.32] | 0.07 | - | - |
| Coil type | | | | | | |
| round | 2 | 34/29 | 5.51 [4.34,6.67] | <0.00001 | 0% | 0.92 |
| figure-of-eight | 2 | 27/25 | 5.01 [-0.02,10.05] | 0.05 | 0% | 0.99 |
| butterfly coil | 1 | 31/32 | 2.00 [-4.91,8.91] | 0.57 | - | - |
| parabola coil | 1 | 16/16 | 1.30 [-5.74,8.34] | 0.72 | - | - |

Results of FMA-UE subgroup analyses
