# Supplementary material for: Efficacy of repeated peripheral magnetic stimulation on upper limb motor function after stroke: a systematic review and meta-analysis of randomized controlled trials
Source: Front Neurol. 2025 Apr 3;16:1513826. doi: 10.3389/fneur.2025.1513826 (PMC12003123; doi:10.3389/fneur.2025.1513826)
Supplement: Supplementary file 3 [file Table_3.DOCX]

**Author(s):** Defu Liao
**Date:** 2025-02-03
**Question:** Should rPMS be used for upper limb motor function after stroke?
**Settings:**
**Bibliography:** Cochrane Database of Systematic Reviews [Year], Issue [Issue].

| **Quality assessment** | | | | | | | **No of patients** | | **Effect** | | **Quality** | **Importance** |  |
| --- | --- | --- | --- | --- | --- | --- | --- | --- | --- | --- | --- | --- | --- |
|  |  |  |  |  |  |  |  |  |  |  |  |  |  |
| **No of studies** | **Design** | **Risk of bias** | **Inconsistency** | **Indirectness** | **Imprecision** | **Other considerations** | **RPMS** | **Control** | **Relative (95% CI)** | **Absolute** |  |  |  |
| **FMA-UE (Better indicated by lower values)** | | | | | | | | | | | | |  |
| 7 | randomised trials | no serious risk of bias | no serious inconsistency | no serious indirectness | serious^1^ | none | 148 | 142 | - | MD 3.34 higher (0.53 to 6.15 higher) | ⊕⊕⊕O MODERATE | CRITICAL |  |
| **FIM (Better indicated by lower values)** | | | | | | | | | | | | |  |
| 2 | randomised trials | no serious risk of bias | no serious inconsistency | very serious^1,2^ | no serious imprecision | none | 54 | 52 | - | MD 0.85 higher (0.19 to 1.51 higher) | ⊕⊕OO LOW | IMPORTANT |  |
| **MAS (Better indicated by lower values)** | | | | | | | | | | | | |  |
| 2 | randomised trials | no serious risk of bias | no serious inconsistency | very serious^1,2^ | no serious imprecision | none | 41 | 27 | - | MD 0.66 lower (1.16 to 0.15 lower) | ⊕⊕OO LOW | IMPORTANT |  |

^1^ The sample size of the included study was too small
^2^ The confidence interval is too wide

Quality of Evidence Chart
