# Supplementary material for: Efficacy of repeated peripheral magnetic stimulation on upper limb motor function after stroke: a systematic review and meta-analysis of randomized controlled trials
Source: Front Neurol. 2025 Apr 3;16:1513826. doi: 10.3389/fneur.2025.1513826 (PMC12003123; doi:10.3389/fneur.2025.1513826)

Forest plot of subgroup analysis

Age


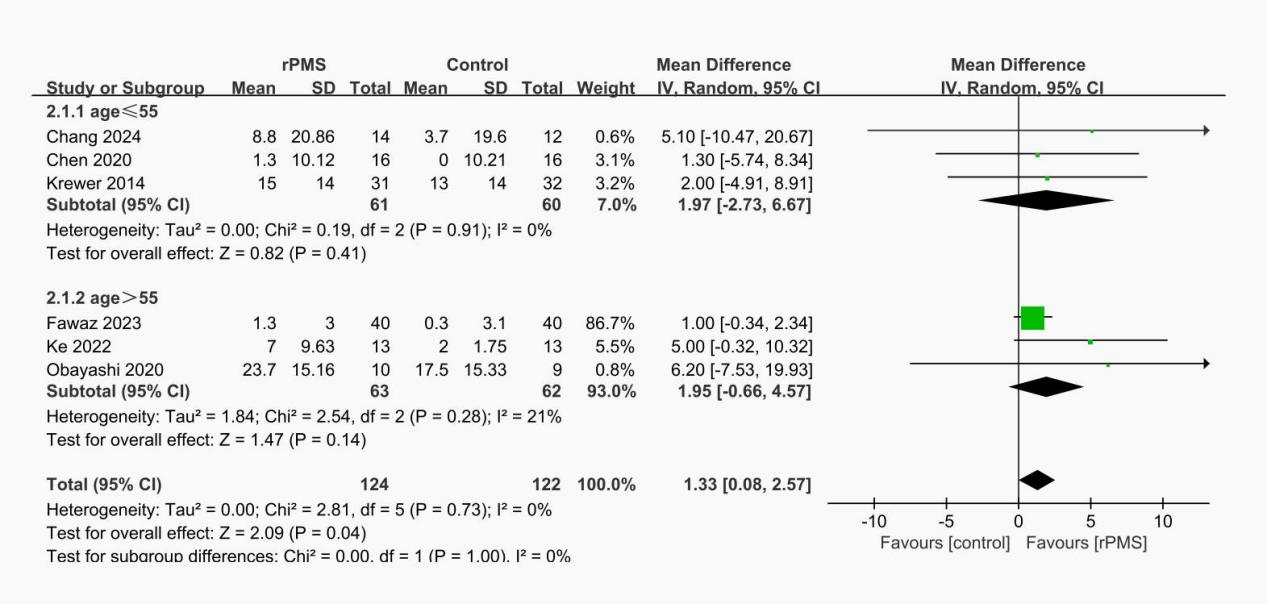


Frequency


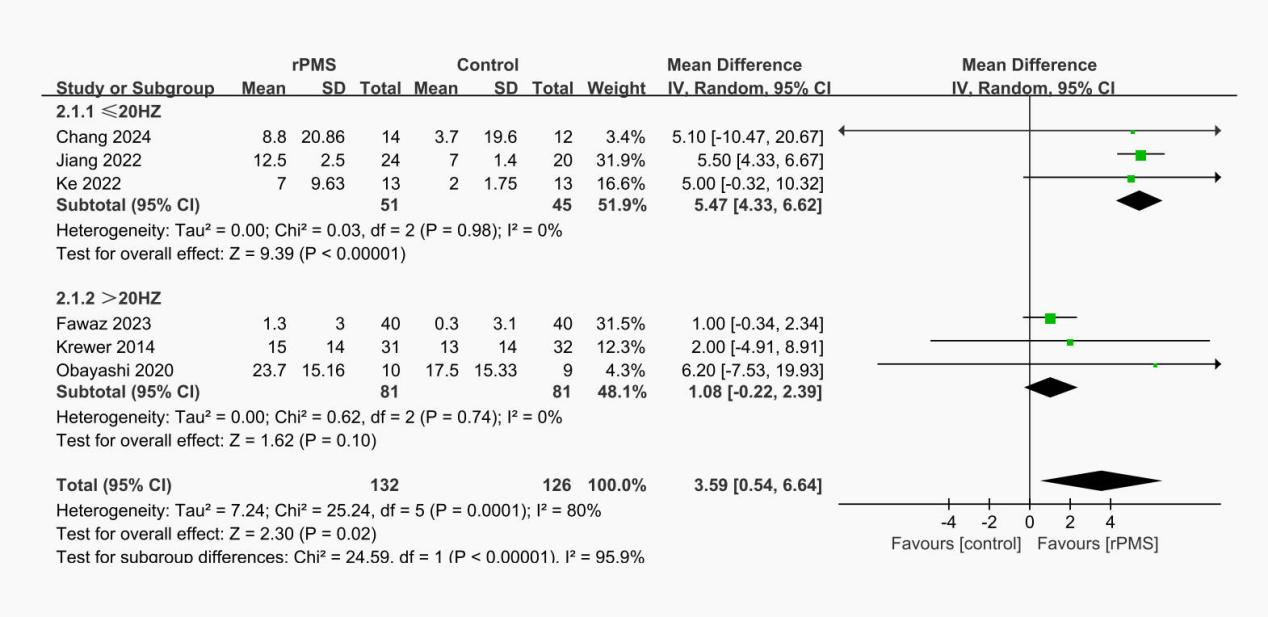


Treatment time


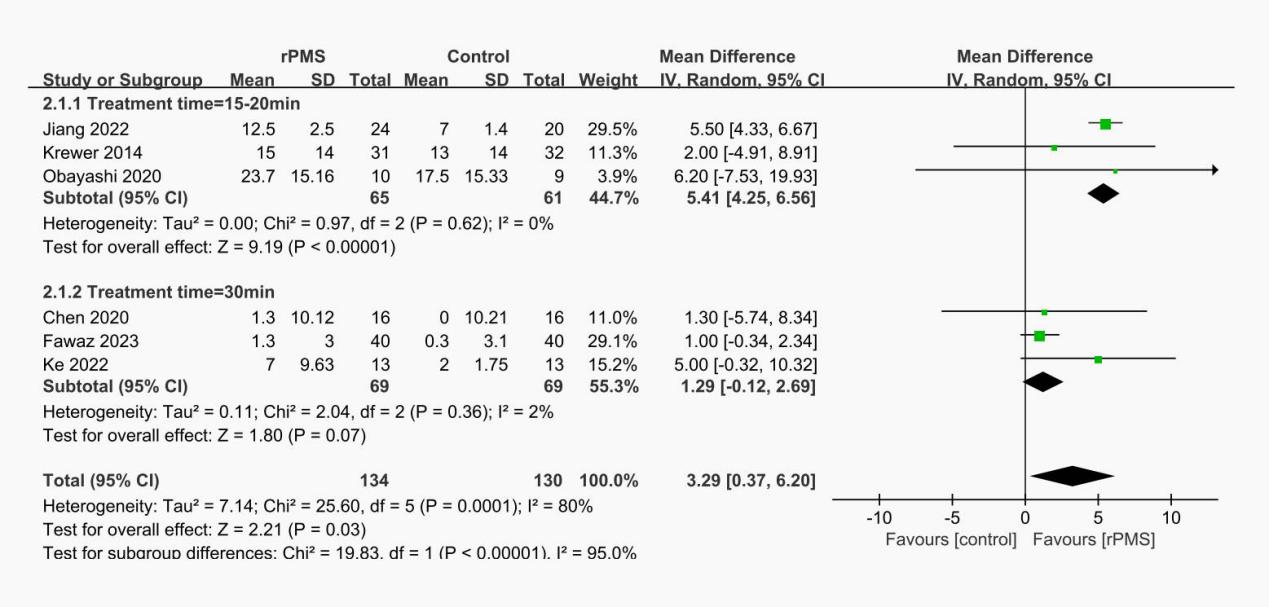


Time post-stroke


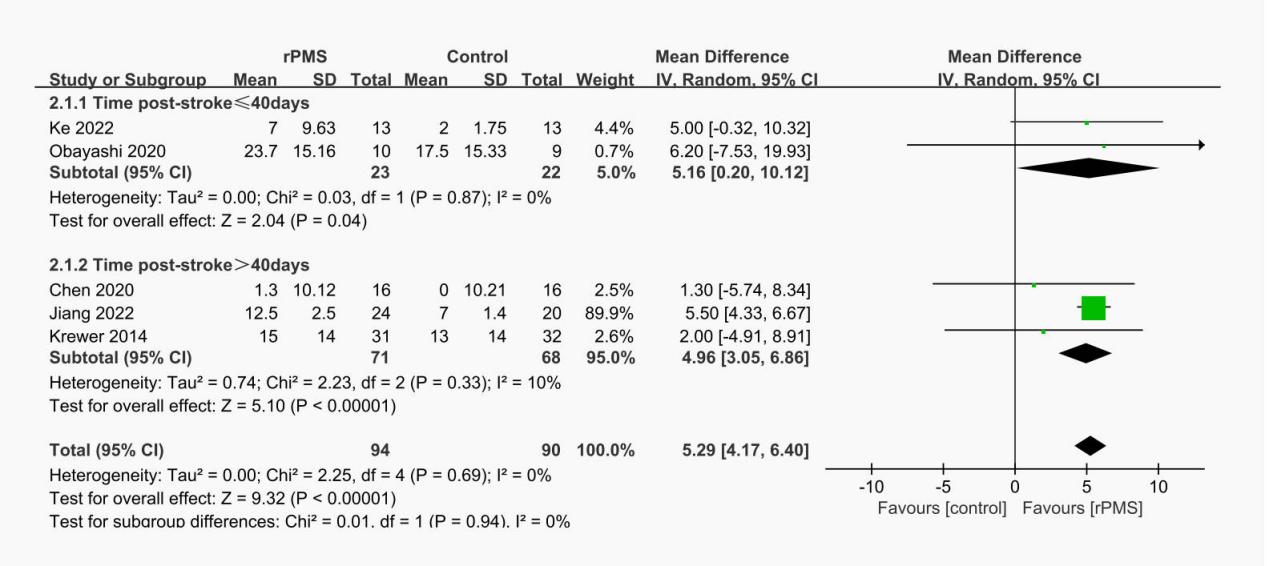


Treatment duration


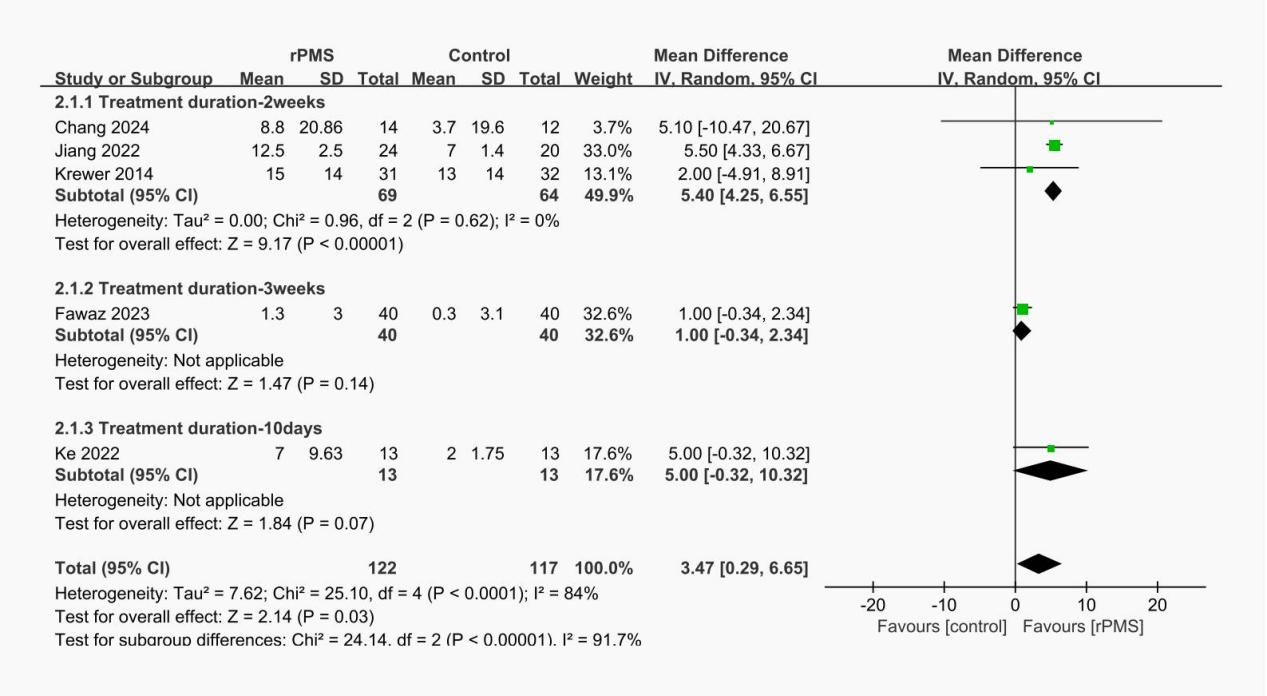


Coil type


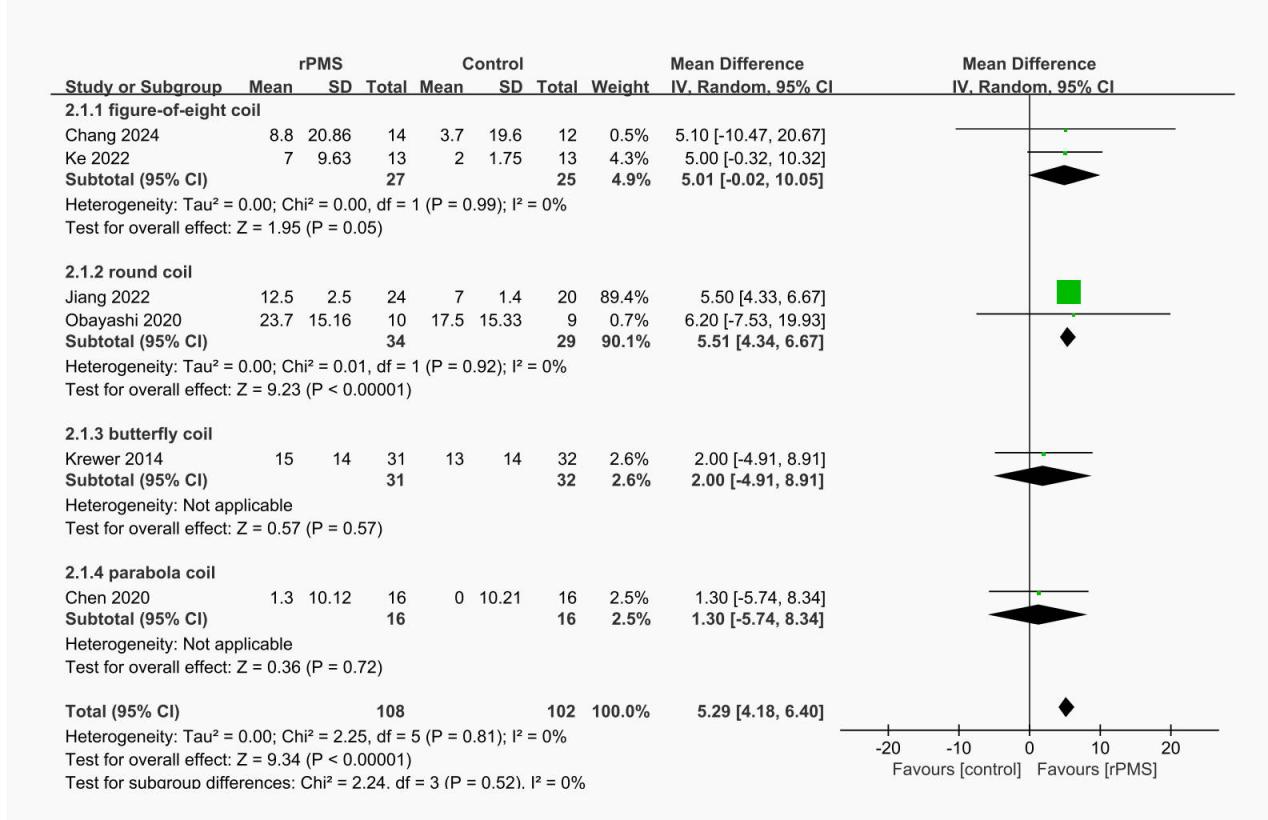

Supplement: Supplementary file 5 [file Table_5.DOCX]
